# Gallic acid protects intervertebral disc cells from ferroptosis and alleviates intervertebral disc degeneration by regulating key factors of oxidative stress

**DOI:** 10.3389/fphar.2025.1501725

**Published:** 2025-02-03

**Authors:** Zaishi Zhu, Zeling Huang, Chaofeng Zhang, Bo Xu, Hua Chen, Shuai Pei, Baofei Zhang, Lishi Jie, Xiaoqing Shi, Yujiang Liu, Yuwei Li, Xiaofeng Shen

**Affiliations:** ^1^ Suzhou TCM Hospital Affiliated to Nanjing University of Chinese Medicine, Suzhou, Jiangsu, China; ^2^ Jiangsu Province Hospital of TCM Affiliated to Nanjing University of Chinese Medicine, Nanjing, Jiangsu, China; ^3^ Orthopaedic Traumatology Institute, Suzhou Academy of Wumen Chinese Medicine, Suzhou, Jiangsu, China

**Keywords:** ferroptosis, intervertebral disc degeneration, oxidative stress, nucleus pulposus, gallic acid

## Abstract

**Background:**

Intervertebral disc degeneration (IDD) is a chronic degenerative disease and one of the main causes of low back pain (LBP). Currently, there is no effective treatment. Ferroptosis is a cell-regulated process that depends on iron deposition and lipid peroxidation. Inhibiting ferroptosis in nucleus pulposus cells is considered a potential strategy for the treatment of IDD. Gallic acid (GA) is naturally present in a variety of plants and has anti-inflammatory, antioxidant and analgesic effects. It has been shown to alleviate ferroptosis. However, the role of GA in IDD ferroptosis remains unclear.

**Methods:**

This study explored the pathological mechanism of GA in IDD in relation to ferroptosis: (1) to identify ferroptosis-related targets for GA treatment of IDD using network pharmacology and molecular docking technology, (2) to evaluate the therapeutic effect of GA in an IDD rat model and changes in ferroptosis-related targets, (3) to investigate the changes of oxidative stress and lipid peroxidation products in NP cells after GA intervention, and (4) to study the changes of ferroptosis-related proteins and iron ions in cells and mitochondria after GA intervention.

**Results:**

Experimental results confirmed that GA can treat IDD by reducing the degradation of extracellular matrix (ECM) and pathological changes in IDD. GA can also mitigate ferroptosis by reducing oxidative stress and lipid peroxidation in rat nucleus pulposus (NP) cells.

**Conclusion:**

The alleviation of disc degeneration ferroptosis by GA may be closely associated with the key ferroptosis proteins P53 and NRF2.

## 1 Introduction

LBP is a prevalent and challenging health issue globally (Sakai and Grad, 2015), with IDD and its associated conditions being among the most frequent causes. IDD seriously affects the quality of life of young and elderly people and brings huge socioeconomic burden (Wu et al., 2020). NP is the gelatinous avascular tissue in the center of the intervertebral disc, mainly composed of NP cells, proteoglycans and type II collagen, and is the most significant target of IDD (Raj, 2008). Under the influence of various pathological factors, NP tissue becomes more fibrous rather than gelatinous, accompanied by the reduction of type II collagen and proteoglycans, ultimately leading to the occurrence and progression of IDD (Antoniou et al., 1996). NP cells’ degeneration is often accompanied by a series of pathological changes such as ECM metabolic imbalance, degradation of type II collagen, downregulation of aggrecan, upregulation of pro-inflammatory cytokines and oxidative stress (Feng et al., 2016; Suyama et al., 2022). At present, the exact molecular mechanism of IDD still needs to be elucidated in detail, but there is evidence that the occurrence process of IDD is the result of the joint action of multiple factors (Oichi et al., 2020).

The role of ferroptosis in IDD has been a research hotspot in recent years (Oichi et al., 2020; Yang et al., 2021; Lu et al., 2021). Ferroptosis is a form of iron- and reactive oxygen species (ROS)-dependent regulated cell death (RCD) that has been shown to be associated with a variety of degenerative diseases (Dixon et al., 2012). It is characterized by iron-dependent lipid peroxidation of cell membranes, unstable iron deposition, excessive ROS production, mitochondrial shrinkage and increased mitochondrial membrane density (Jiang et al., 2021). Cells or tissues may undergo ferroptosis when iron-induced reactive oxygen species cause oxidative damage (Stockwell et al., 2017). Increasing evidence suggests that ferroptosis affects IDD by reducing the viability of NP cells, annulus fibrosus cells, or endplate chondrocytes and increasing the degradation of the ECM (Feng et al., 2016; Zhang et al., 2023; Wang et al., 2022). Due to similar pathological processes, oxidative stress is closely related to ferroptosis (Dixon and Stockwell, 2014), and the crosstalk between oxidative stress and ferroptosis has been studied in several diseases (Li et al., 2022). However, the relevance of the crosstalk between oxidative stress and ferroptosis in IDD has not been fully explored.

GA is a triphenolic compound that can be extracted from Polygonum cuspidatu (Ma et al., 2015), Forsythia suspensa (Wang et al., 2018), and gallnut. GA has a wide range of applications in food, biology, medicine and chemicals, and is often used as a dietary supplement or additive with clinical significance. It has anti-cancer, anti-inflammatory, and anti-oxidative stress effects in various diseases (Wang et al., 2018). In recent years, there has been some recognition of research on GA ferroptosis (Tang and Cheung, 2019). Studies have shown that GA can regulate the P2X7/ROS signaling pathway, effectively reduce the level of oxidative stress in the body, thereby reducing iron death in rat spinal microglia to relieve pain (Yang et al., 2023), and can regulate ferroptosis-related genes to improve colon cancer (Hong et al., 2021). GA has also been reported in the treatment of IDD. Study has shown that GA can reduce the expression of ADAMTS-4 in NP cells by regulating the phosphorylation and acetylation of p65 in the NF-κB signaling pathway (Huang et al., 2017). In addition, GA has also been developed as a new material to alleviate IDD by alleviating oxidative stress and mitochondrial dysfunction (Chen et al., 2024). However, the therapeutic effect of GA on ferroptosis in IDD has not been clarified. Therefore, this study explored the pathological mechanism of GA related to ferroptosis in IDD.

This study aimed to investigate the effect of GA in IDD in relation to ferroptosis. (1) The ferroptosis-related targets were identified for GA treatment of IDD using network pharmacology and molecular docking technology (2) based on pathological staining, immunohistochemistry, and Western blot results, the therapeutic effects of GA and its impact on ferroptosis-related indicators were verified in IDD rat models, and (3) the mechanism of GA in mitigating IDD ferroptosis was verified by detecting iron ions in cells and mitochondria and detecting ROS and Liperfluo. These findings contribute to the development of GA as a novel therapy for IDD and provide evidence of its association with ferroptosis.

## 2 Methods and materials

### 2.1 Network pharmacology analysis

Obtain GA-related targets in the TCMSP, STITCH, BatmanTCM, SwissTargetPrediction, Symmap, TCMIP and ChEMBL databases. Conduct target searches in the GeneCard, DisGeNET, and MalaCards databases using “Intervertebral disc degeneration” as the search term. The IDD-related targets found in the three databases were downloaded and then duplicated and intersected. Download ferroptosis-related targets in the ferroptosis database (http://www.zhounan.org/ferrdb/current/). The targets at the intersection of the three were identified as potential targets related to ferroptosis in GA treatment of IDD. The protein-protein interaction (PPI) network for the potential targets was established using the String database. Cytoscape 3.7.2 software was employed to construct the visual representation of the PPI network and to identify core targets based on the network’s Betweenness Centrality values.

### 2.2 Molecular docking

The 3D structure of TP53 (PDB ID: 6MY0), PPARG (PDB ID: 6C5Q), NFE2L2 (PDB ID: 2FLU), JUN (PDB ID: 5FV8) were downloaded from the PDB database (https://www.rcsb.org/), and the GA component structure was downloaded from the PubChem database (https://pubchem.ncbi.nlm.nih.gov/). The water molecules and small molecule ligands in the key target receptor structure were removed using PyMOL2.4.1 software, and then imported into AutoDockTools1.5.6 software to select the docking site centered on the original ligand and set the girdbox. The molecular docking was completed using AutoDockVina software, and the docking results were output as txt files and pdbqt files. The minimum binding energy of the two was viewed through the txt file, and the pdbqt file was opened with PyMOL2.4.1 to view the docking image.

### 2.3 Animals

Male Sprague–Dawley rats weighing 200–220 g were obtained from the Animal Center of the Nanjing University of Traditional Chinese Medicine. The rats were bred and housed in the Experimental Animal Center of Nanjing University of Traditional Chinese Medicine under license number (Jiangsu): No.202306A054. The raising conditions included a room temperature of 22°C–26°C and a relative humidity of 40%–70%. They were treated according to the National Institutes of Health Guidelines for the Care and Use of Laboratory Animals. All experiments were approved and supervised by the Animal Care and Use Committee of the Nanjing University of Traditional Chinese Medicine. This study was reviewed and approved by the Animal Care and Use Committee of the Nanjing University of TCM (Approval number: No. 202306A054).

### 2.4 IDD rat model

Fifty male Sprague-Dawley rats (aged 6 weeks) were housed in a specific pathogen-free animal laboratory (humidity, 60%–65%; temperature, 22°C–25°C), with free access to food and water under a 12-h light and dark cycle. The experiment began after 1 week of acclimation. Among them, 40 rats were randomly selected and injected with pentobarbital (1 mL/kg) for general anesthesia. After alcohol disinfection, the 21 G needle was used to puncture the caudal disc at the Co6/7/8 segment of the tail, and the puncture was 5 mm toward the center of the disc. After puncture, the needle was rotated 360° and left in place for 10 s (Han et al., 2008). For IDD treatment, GA was administered orally, and the dose was calculated based on previous studies using the dosage conversion formula between humans and rats. Equivalent dose coefficient conversion formula, the low dose for rats was 25 mg/kg, the medium dose was 50 mg/kg, and the high dose was 100 mg/kg. The sham operation group and the model group were intragastrically administered with an equal volume of normal saline. After four consecutive weeks, intervertebral disc tissue was collected for further histological analysis.

### 2.5 X analysis

Place the tail of the rat on the X-ray photography equipment, and perform X-ray photography under the conditions that the distance from the collimator to the film is 66 cm, the penetration power is 35 kV, and the exposure time is 63 ms (Keorochana et al., 2010). Use ImageJ software to measure the intervertebral space and vertebral body height, and calculate the intervertebral disc height index (DHI). The intervertebral disc height index = the sum of the intervertebral space height/the height of adjacent vertebral bodies (Han et al., 2008).

### 2.6 Hematoxylin and eosin, safranin O/fast green, masson and toluidine blue’s staining

The intervertebral disc tissue was fixed with 4% paraformaldehyde and then decalcified, and the tissue blocks were embedded in paraffin. The embedded tissue sections were routinely deparaffinized and stained with hematoxylin and eosin (HE), Safranin O/fast green (SO/FG), Masson and Toluidine Blue (TB) sequentially, following the instructions provided with the staining kit (Sorabio, China). The morphological structure was examined using a digital microscope (IX-71, Olympus, Japan).

### 2.7 Immunohistochemistry detection

Paraffin-embedded the intervertebral tissue samples (4 mm thick) were prepared and subjected to antigen retrieval. After that, the sections were incubated overnight at 4°C with the following primary antibodies: MMP3 rabbit antibody (1:100; number PA5-34737; Proteintech, China), ADATMS4 rabbit antibody (1:100; ab287968; Proteintech, China). P53 (1:100, Proteintech, China), and Nrf2 (1:100, Proteintech, China). The sections were incubated with biotinylated goat anti-rabbit IgG (1:2500; ab205718; Abcam Inc.) for 20 min, and then with horseradish peroxidaseestreptavidin reagent for 20 min. Finally, the immunoreactivity was detected using diaminobenzidine, and the sections were counterstained with hematoxylin. Images were obtained using a microscope (IX-71, Olympus, Japan). Quantitative analysis was performed using ImageJ software.

### 2.8 Nucleus pulposus primary cell culture

Gelatinous NP tissue from 4-week-old normal male Sprague-Dawley rats was digested with 0.2% type II collagenase (Sigma, St. Louis, MO, United States) 2 h. The cells were then cultured in complete medium (DMEM/F12, Gibco, Invitrogen, United States) containing 10% fetal bovine serum (FBS, Corning, United States) and antibiotics in an environment of 5% CO_2_ and 37°C. Change culture medium every 2–3 days.

### 2.9 Transmission electron microscope for assessment of mitochondrial damage

Collect the cells in 5 mL Eppendorf tubes. Slowly add 2% glutaraldehyde fixative precooled to 4°C into 5 mL Eppendorf tubes for fixation. Dehydrate with gradient acetone and embed with epoxy resin 618. After embedding, stain with uranyl acetate and lead citrate. Transmission electron microscopy (JEM1010, JEOL, Tokyo, Japan) was used to observe the changes in mitochondrial ultrastructure and evaluate the changes and damage of ultrastructure.

### 2.10 Immunofluorescence detection

The localization and content changes of MMP3 rabbit antibody (1:100; 17873-1-AP; Proteintech, China), Collagen Tybe Ⅱ rabbit antibody (1:100; 28459-1-AP; Proteintech, China). In the subchondral bone were detected using the immunofluorescence method. ImageJ was used to detect the intensity and area of the corresponding fluorescence, and we counted the number of colocalizations.

### 2.11 ROS detection

Use a 6-well plate to inoculate NP cells, and add drugs after they adhere to the wall and grow to about 70%. After 24 h of intervention, perform ROS detection. Dilute the Loading Buffer according to the instructions of the kit (R253, Dojindo, Japan) and prepare the working solution. Remove the culture medium and wash the cells three times with PBS. Remove the supernatant, add the prepared working solution, and culture in a 37°C, 5% CO_2_ incubator for 30 min and then washed three times with PBS. After adding PBS again, detect fluorescence.

### 2.12 Liperfluo detection and the level of MDA

NP cells were seeded in 6-well plates at a density of 4 × 10^5^ cells per well. The cells were treated according to different groups for 24 h. Then prepared the working solution according to the instructions of the kit (L248, Dojindo, Japan). The cells were incubated at 37°C in the dark for 30 min with the working solution containing 1 mM Liperfluo solution. After washing with PBS three times, the lipid peroxide level was observed under a fluorescence microscope in the dark. ImageJ software was used for quantitative analysis. The level of malondialdehyde (MDA) was determined using MDA kits (A003-1, Nanjing Jiancheng, China).

### 2.13 Detection of Mito-FerroGreen and FerroOrange

We used FerroOrange (F374, Dojindo, Japan) and Mito-FerroGreen (M489, Dojindo, Japan) to characterize ferroptosis in nucleus pulposus cells. NP cells were processed in 6-well plates and then washed three times with PBS. Mito-FerroGreen was then dissolved in dimethyl sulfoxide (DMSO, Solarbio, China) as a working reagent. NP cells were then incubated with the prepared working reagent at a concentration of 1 μmol/L FerroOrange or 5 μmol/L Mito-FerroGreen for 30 min at 37°C in the dark, and then washed three times with PBS. Fluorescence images were finally obtained using a fluorescence microscope (Olympus, Japan).

### 2.14 Cell counting Kit-8 (CCK-8) assay

NP Cells in the logarithmic growth phase were seeded into 96-well plates (1 × 10^5^ cells per well) and incubated at 37°C in a 5% CO_2_ incubator. After experimental treatment for 24 h, CCK-8 reagent (10 μL, Beyotime, China) was added, and the cells were incubated. The cell viability of each group was tested by measuring the absorbance (450 nm) using a microplate reader.

### 2.15 Western blot analysis

Weigh the NP tissue, add RIPA lysis buffer at a standard of 100 mg/200 μL. Homogenize the mixture for 5 min and then centrifuge at 4°C for 30 min to obtain the supernatant. Protein concentrations were determined using the BCA assay. Based on the protein concentration, samples were prepared for electrophoresis, with bromophenol blue added to the bottom of the gel. After electrophoresis, the proteins were wet-transferred onto a PVDF membrane. The membrane was washed with TBST and subsequently blocked with 5% skim milk powder at room temperature. Standard Western blot analysis was performed to detect the protein expression of GPX4 (1:1,000, 67763-1-Ig, Proteintech, China), ACSL4 (1:1,000, 22401-1-AP, Proteintech, China), P53 (1:1,000, 60283-2-Ig, Proteintech, China), HO-1 (1:1,000, 10701-1-AP, Proteintech, China), and Nrf2 (1:1,000, 16396-1-AP, Proteintech, China). β-Actin (1:1,000, 66009-1-Ig, Proteintech, China) was used as the reference protein.

### 2.16 Data analysis

The research results were statistically processed using the Statistical Package for the Social Sciences (SPSS) 25.0 software, and GraphPad Prism 8.0 software was used for statistical graph processing.

## 3 Results

### 3.1 Network pharmacology prediction and molecular docking verification

**FIGURE 1:**
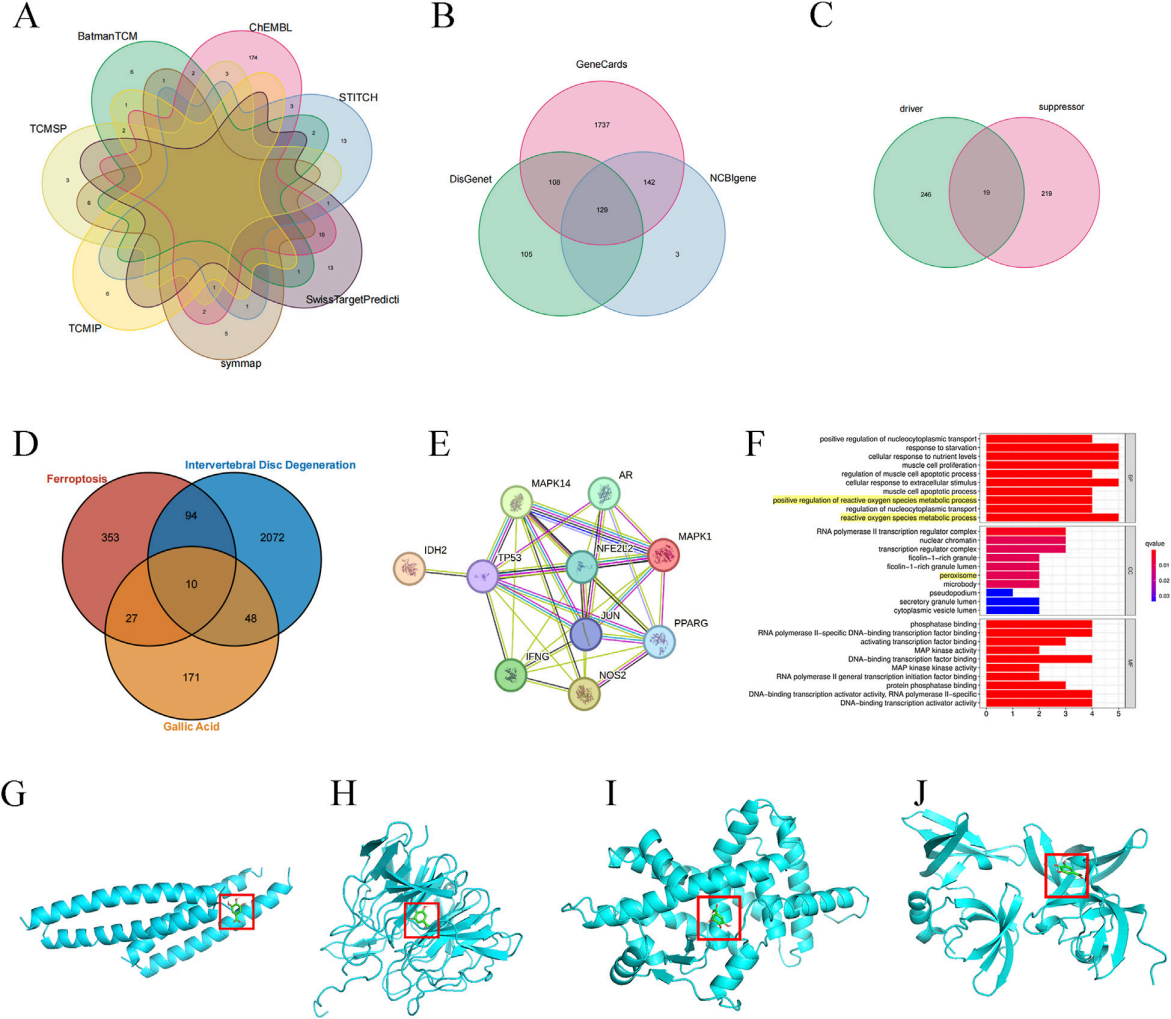
Prediction of targets related to ferroptosis in IDD alleviated by GA. **(A)** GA targets map **(B)** IDD targets map **(C)** Ferroptosis-related targets **(D)** GA, IDD and ferroptosis target Venn diagram **(E)** PPI Network Diagram. **(F)** Target gene function enrichment analysis. **(G–J)** JUN, NFE2L2, PPARg, TP53 and GA docking verification results display.

256 GA targets were collected from 7 databases by removing duplicates (Figure 1A). Obtain IDD -related expression genes from the Genecards database, NCBIgene database and Disgenet database. Use R language to perform gene deletion and union of three databases. Among them, there are 2166 related genes and 429 duplicated genes in the Genecards database; there are 274 related genes and 271 duplicated genes in the NCBIgene database; and there are 342 related genes and 237 duplicated genes in the Disgenet database. Finally, 2224 IDD genes were obtained (Figure 1B). A total of 484 ferroptosis-related targets were obtained in the ferroptosis database (Figure 1C). Through the Venn website (http://bioinformatics.psb.ugent.be/webtools/Venn/), the obtained target genes of GA active ingredients, IDD-related genes and ferroptosis-related targets were intersected, and the results of the three were obtained. Common target genes (Figure 1D). These intersection genes are TP53, MAPK1, MAPK14, IFNG, PPARG, NFE2L2, JUN, IDH2, NOS2, AR. Import these genes into the PPI network for network topological heterogeneity analysis (Figure 1E). The DC, BC, and CC information of the ten targets are shown in Table 1. GO functional enrichment analysis of biological processes suggests that biological processes are mainly enriched in oxygen species metabolic process and peroxisome (Figure 1F).

**TABLE 1:** Targets information.

Targets	BC	CC	DC
TP53	17.633333	1.0	9
PPARG	1.6333333	0.9	8
JUN	1.6333333	0.9	8
NFE2L2	1.6333333	0.9	8
MAPK1	0.8	0.8181818	7
IFNG	0.33333334	0.8181818	7
MAPK14	0.33333334	0.8181818	7
NOS2	0.0	0.75	6
AR	0.0	0.6923077	5
IDH2	0.0	0.5294118	1

Docking simulation is a convenient and effective method to explore the interaction between small molecules and targets. Here, AutoDockVina1.1.2 software is used to simulate the docking of small molecule GA with the top-ranked TP53, PPARG, NFE2L2, and JUN proteins, and obtain their binding energy scores. Negative values for binding energy indicate the likelihood of binding. In general, values less than −5 kcal/mol are considered more likely to bind. In this study, the binding affinity of GA to TP53, PPARG and NFE2L2 proteins was −5.6 kcal/mol, −5.6 kcal/mol and −7.0 kcal/mol (Table 2), indicating that small molecule GA has ideal binding to TP53, NFE2L2, and PPARg protein’s ability (Figures 1G–J).

**TABLE 2:** Binding energy of core target binding to GA.

Targets	Active ingredient	Binding energy/(kcal/mol)
TP53	GA	−5.6
PPARG	GA	−5.6
NFE2L2	GA	−7.0
JUN	GA	−4.7

### 3.2 GA can effectively alleviate intervertebral disc degeneration in rat models

**FIGURE 2:**
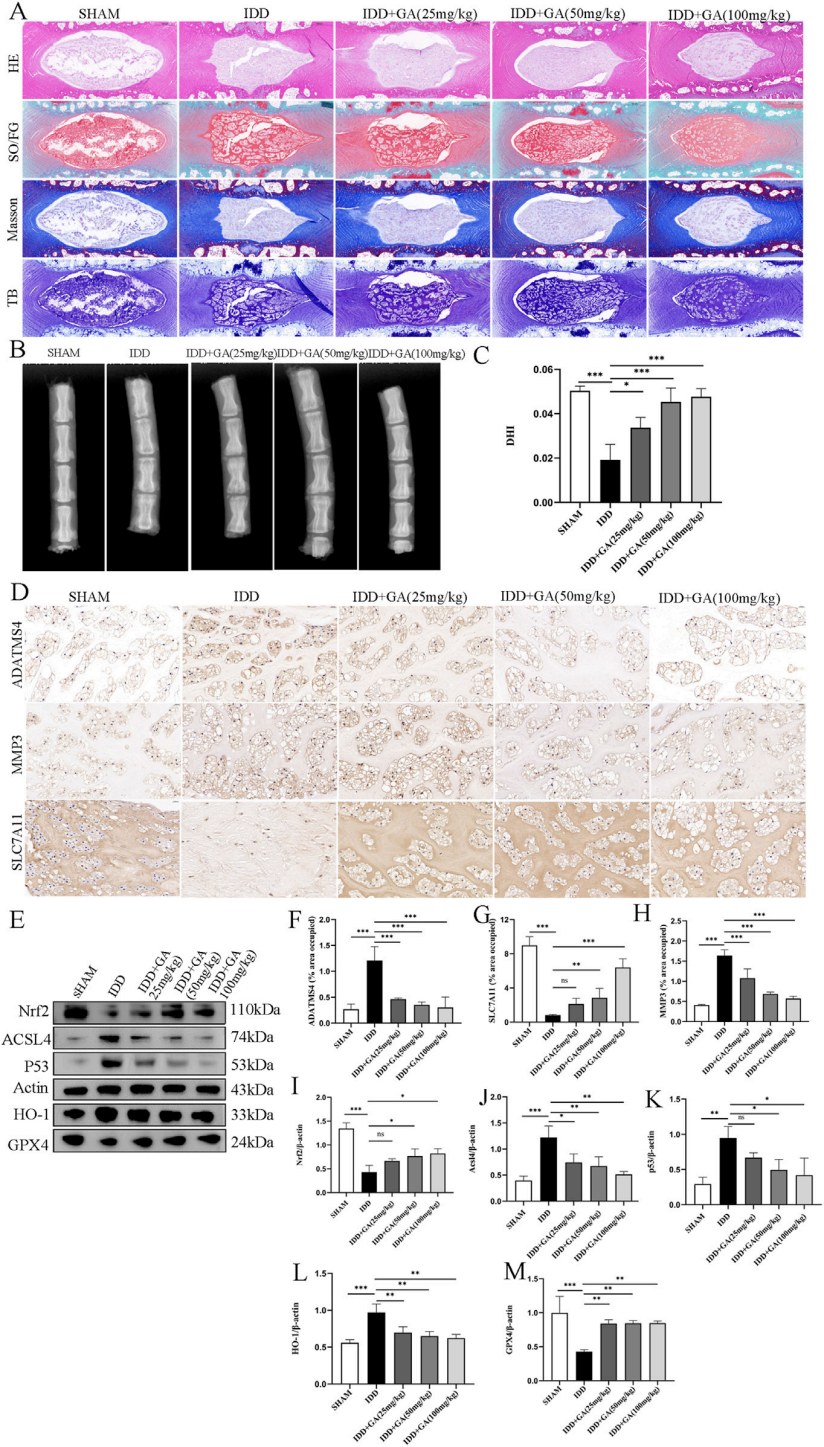
The efficacy of GA and evidence of its improvement in ferroptosis. **(A)** HE, SO/FG, Masson and TB staining of morphological changes of intervertebral disc tissue. **(B)** X-ray of rat caudal vertebrae showed that the intervertebral space collapsed in the needle-punctured group. After intervention with GA at different concentrations, the results showed a significant improvement. **(C)** DHI of rat caudal vertebrae. The intervertebral space was measured using ImageJ to calculate DHI. **(D)** Representative images (scale bar = 20 μm) of immunohistochemical staining of ADATMS4, MMP3 and SLC7A11 proteins of intervertebral disc. **(E)** Representative images of ferroptosisrelated proteins in NP. **(F–H)** Semi-quantitative histogram of immunohistochemical staining of ADATMS4, MMP3 and SLC7A11 proteins of intervertebral disc. **(I–M)** Quantitative analysis of ferroptosisrelated proteins in NP.

The rat tail intervertebral disc puncture model is a commonly used animal model in intervertebral disc research. Punctured discs were treated with 25 mg/kg, 50 mg/kg and 100 mg/kg GA for 4 weeks. We performed X-rays to evaluate NP degeneration. Intervertebral disc height measurement and DHI calculation showed that the degenerated disc height decreased by more than 50%, and the disc height in the treatment group decreased less, returning to about 80% of the sham operation group (Figures 2B, C). The results of HE staining, Safranin O-Fast Green staining, Masson staining and TB staining showed that there was no obvious shrinkage of the NP in the intervertebral disc tissue of the rats in the sham operation group, the reticular structure was intact, the boundaries with the annulus fibrosus were clear, and the annulus fibrosus was arranged orderly; Rats in the IDD group show that the NP in the intervertebral disc tissue is shrunken and structurally disordered, the boundaries between the annulus fibrosus of NP are unclear, and the annulus fibrosus is partially torn and arranged disorderly. The NP in the intervertebral disc tissue of rats in the GA low-dose group (25 mg/kg) and the medium-dose group (50 mg/kg) shrank by more than half, and the structure was disordered. The boundary with the annulus fibrosus was still clear, and the annulus fibrosus structure was still intact, with serpentine patterns appearing. The NP in the intervertebral disc tissue of rats in the GA high-dose group (100 mg/kg) is partially shrunken and structurally disordered, with a clear boundary with the annulus fibrosus, and the annulus fibrosus structure is still intact and arranged normally (Figure 2A). ADATMS4 and MMP3 immunohistochemistry results suggested that the expression of MMP3 and ADATMS4 increased in the IDD rat model, and their expression decreased after GA treatment. In addition, SLC7A11 immunohistochemistry result showed that GA treatment can increased the expression of SLC7A11, which was decreased in the IDD rat model. These findings can be used as evidence that GA effectively treats IDD (Figures 2D–G). To further verify the role of ferroptosis in IDD and the effect of GA, Western blot was performed on NP tissue. All three treatment groups at three concentrations significantly increased the expression of NRF2, GPX4 and decreased the expression of P53, ACSL4 and HO-1. The expression of NRF2, GPX4 was significantly increased and the expression of P53, ACSL4 and HO-1 was significantly decreased in the high-dose GA treatment group (100 mg/kg) (Figures 2H–M). These data demonstrate the potential of GA in IDD treatment and its association with ferroptosis.

### 3.3 Oxidative stress and ferroptosis affect the expression of TP53 and NRF2

**FIGURE 3:**
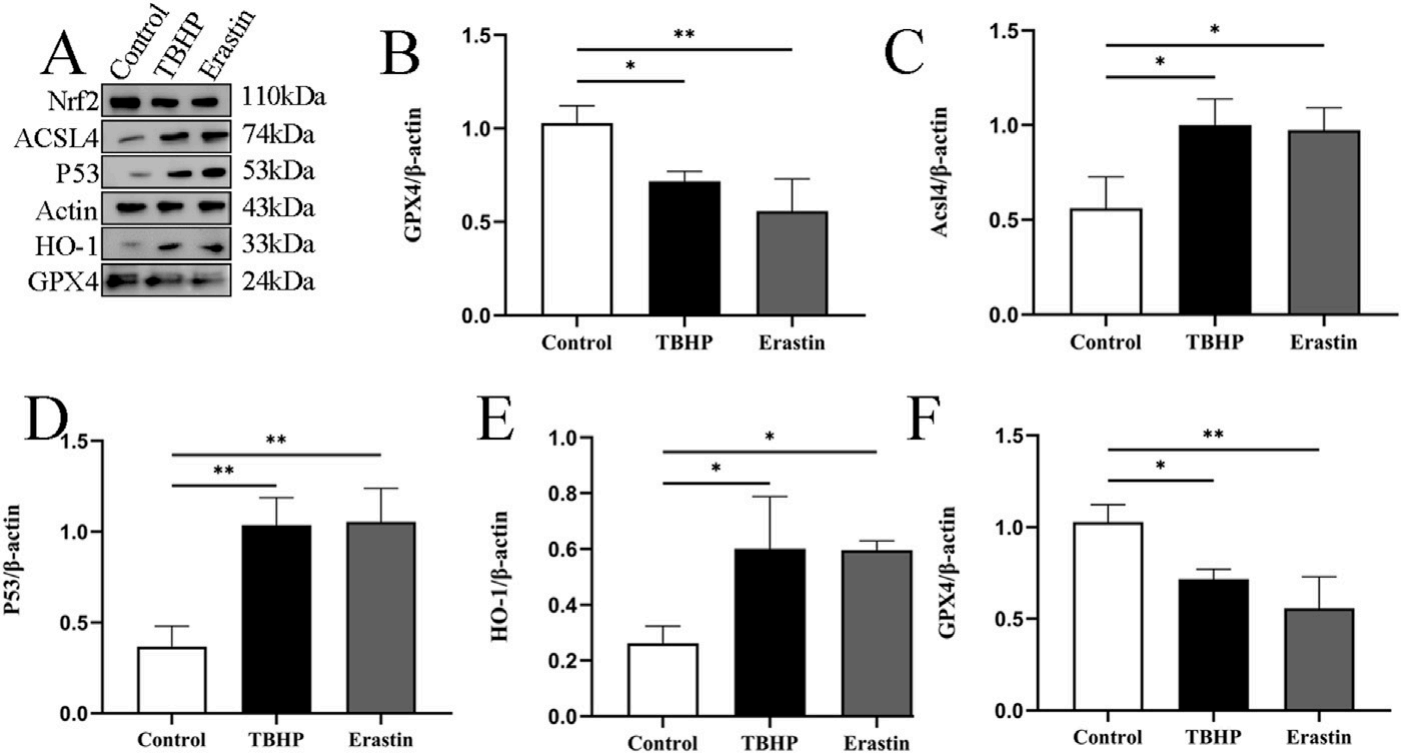
TBHP promotes ferroptosis of NP cells. **(A–F)** Representative images and quantitative analysis of ferroptosis-related proteins after TBHP and Erastin treatment in NP cells. *p < 0.05, **p < 0.01.

Erastin has been shown to induce ferroptosis through multiple pathways. Tert-butyl hydroperoxide (TBHP) has been shown to play an important role in the initiation and progression of IDD by activating oxidative stress and inflammatory responses (Ingold et al., 2018). Referring to the previous method (Ingold et al., 2018), we chose 100 μM TBHP to simulate the conditions around the rat NPC for 24 h. Erastin has also been selected for NP cell intervention. Western blotting results showed that the expression of P53, HO-1, and ACSL4 proteins increased, while the expression of NRF2 and GPX4 proteins decreased (Figures 3A–F).

### 3.4 GA ameliorated ECM degradation in rat NP cells induced by TBHP

**FIGURE 4:**
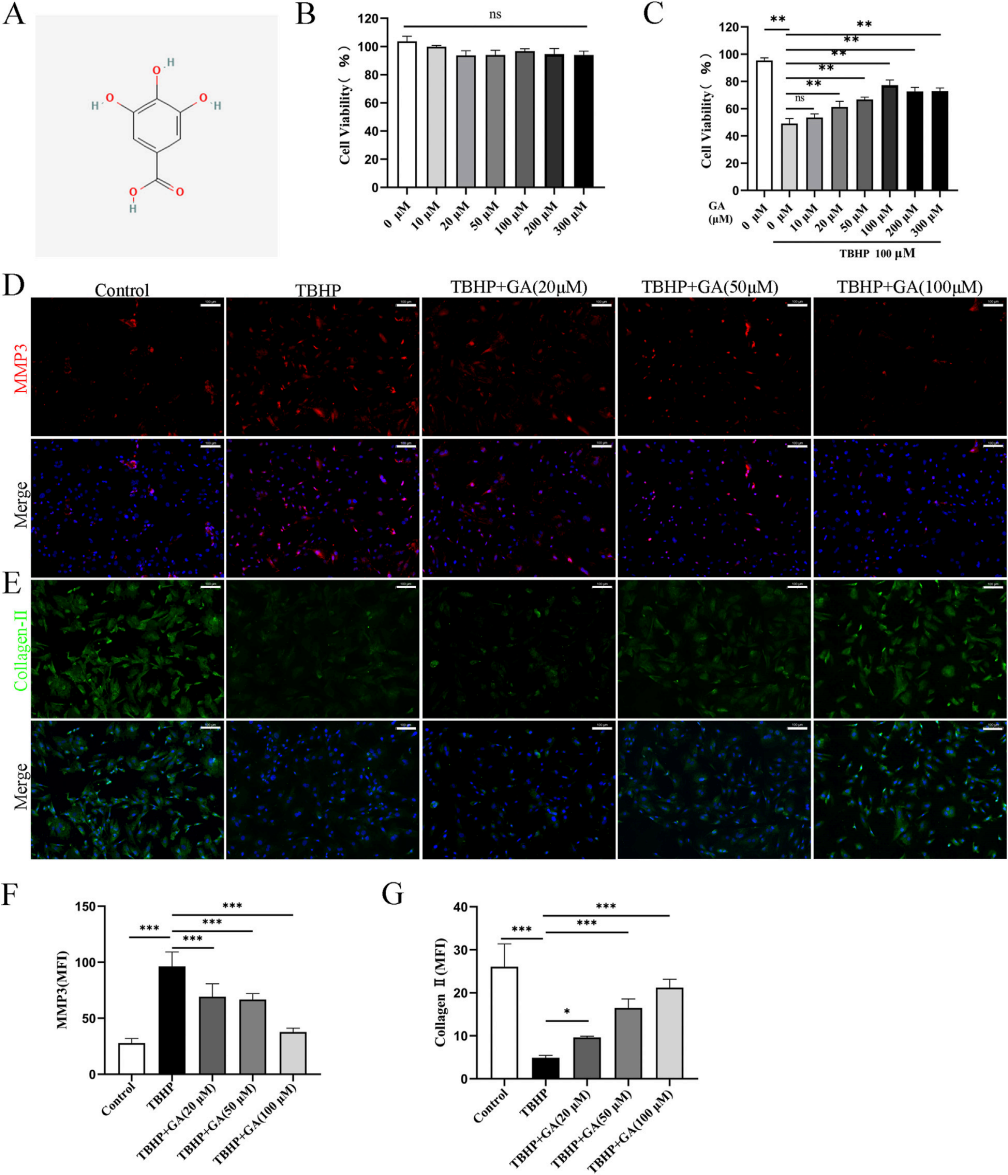
GA promotes ECM secretion in NP cells. **(A)** GA molecular structure. **(B)** Changes in NP cell viability under the intervention of different concentrations of GA **(C)** NP cells’ viability under GA intervention concentration and 100 μm TBHP simulating. **(D–G)** Representative immunofluorescence images (scale bar = 100 μm) of ECM proteins Collagen-Ⅱ and MMP3, along with quantitative analysis of the mean fluorescence intensity in NP cells. *p < 0.05, **p < 0.01, ***p < 0.001, ns for no significance.

The molecular structure of the drug is shown in Figure 4A. Previous studies have shown that peroxide-induced cell death is associated with ferroptosis. IDD is characterized by ECM degeneration (Risbud and Shapiro, 2014). In order to determine the direct biological effect of GA on NP cells, different concentrations of GA (0, 10, 20, 50, 100, 200, 300 μM) were added to NP cells, and then the cell viability was detected using the CCK-8 method. The results showed that GA was no obvious cytotoxicity at concentrations below 300 μM (Figure 4B). After TBHP (100 μM) inhibited 24 h, CCK-8 assay determines the proliferation of NP cells after intervention with different concentrations of GA. The results showed that after 100 μM TBHP intervention, the cell proliferation rate decreased. 10 μM GA had no significant effect on cell proliferation, while 20, 50, and 100 μM GA interventions improved cell proliferation. In addition, compared with 100 μM GA intervention, 200 and 300 μM GA interventions did not further improve cell proliferation rates (Figure 4C). We further explored the impact of GA on TBHP-induced NP cells dysfunction by examining ECM changes. As shown in Figures 4D–G, the expression of Collagen type II in NP cells treated with TBHP (100 μM) was significantly inhibited, but the expression of MMP3 was increased. However, treatment of GA reversed this change.

### 3.5 GA inhibited oxidative stress and lipid peroxidation in NP cells

**FIGURE 5:**
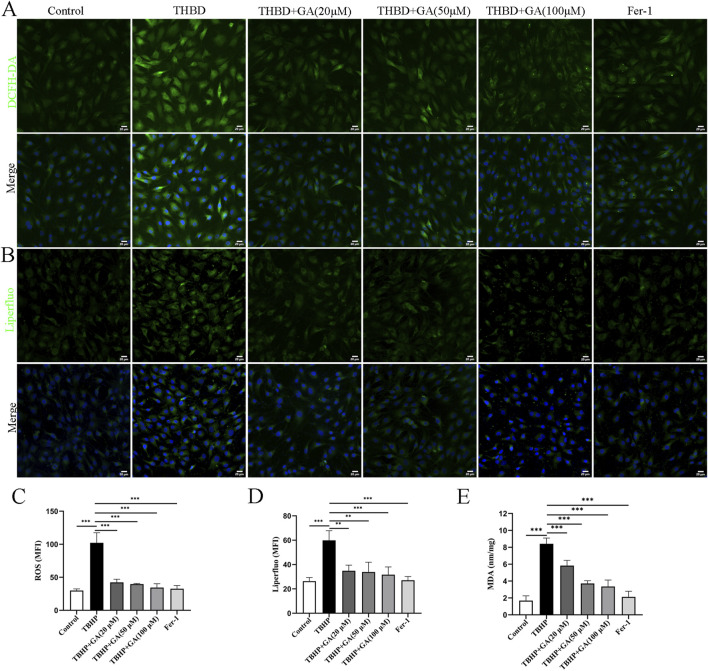
GA mitigates oxidative stress and lipid peroxidation in NP cells. **(A, B)** Representative immunofluorescence images of ROS and Liperfluo in NP cells (scale bar = 20 μm). **(C, D)** Quantitative analysis of the mean fluorescence intensity was quantified using ImageJ. **(E)** ELISA results showed that the MDA content. **p < 0.01, ***p < 0.001.

We further elucidated the mechanism related to GA’s treatment of ferroptosis in NP cells. Ferroptosis is a newly discovered cell death mode accompanied by oxidative stress, and oxidative stress caused by TBHP is an important cause of cellular ferroptosis. The accumulation of lipid peroxidation is also one of the important characteristics of ferroptosis. When the lipid peroxidation in cells increase, it will cause cell membrane damage and lead to cell death (Risbud and Shapiro, 2014). The detection of ROS and lipid peroxides also confirmed the increase in oxidative stress and lipid peroxides induced by TBHP treatment (Figures 5A, B), which was downregulated in a dose-response manner after GA treatment. The average fluorescence intensity statistics are shown in Figures 5C, D. MDA is a metabolite of lipid peroxidation and one of the markers of ferroptosis. MDA increases after TBHP stimulation, and GA treatment can significantly reduce MDA levels (Figure 5E).

### 3.6 GA ameliorated NP cells ferroptosis by regulating the P53/SLC7A11/GPX4 and NRF2/HO-1 signaling pathway

**FIGURE 6:**
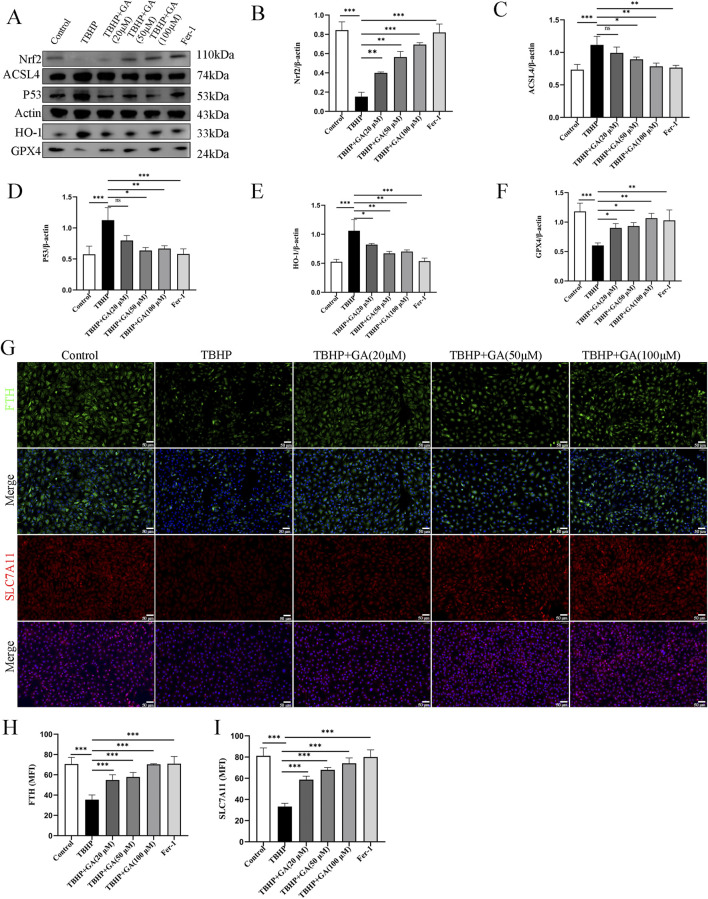
GA can improve the changes of ferroptosis-related proteins induced by TBHP. **(A–F)** Representative images and quantitative analysis of ferroptosis-related proteins in NP cells. **(G–I)** Representative immunofluorescence images (scale bar = 50 μm) and quantitative analysis of the mean fluorescence intensity of SLC7A11 and FTH-1 in NP cells. *p < 0.05, **p < 0.01, ***p < 0.001, ns for no significance.

SLC7A11 and GPX4 are considered recognized ferroptosis biomarkers, and they are relevant downstream regulators of ferroptosis. Nuclear factor E2-related factor 2 (NRF2) is a major regulator of antioxidant and cytoprotective pathways, and GSH-GPX4 is its downstream target in maintaining cellular redox balance (Dodson et al., 2019). NRF2 can regulate intracellular iron metabolism and is associated with oxidative stress and ferroptosis in macrophages (Zhang et al., 2021). NRF2 can also inhibit ferroptosis by regulating SLC7A11 (Dong et al., 2021). TP53 regulates ferroptosis through transcriptional or post-translational mechanisms and exhibits an inhibitory effect on SLC7A11 (Kang et al., 2019). Excess P53 can regulate the expression of its downstream targets and promote the occurrence of ferroptosis. P53 and NRF2 are upstream regulatory genes that act on SLC7A11 (Xie et al., 2023). HO-1 could be inducible and participate in the synthesis of GPX4 (Yang W. et al., 2022). Ferroptosis-related factor levels were examined. TBHP treatment increased the levels of, HO-1 ACSL4, and P53 and decreased those of GPX4, NRF2 compared to those in the Control group. However, GA reversed this effect; HO-1 ACSL4, and P53 levels decreased, whereas GPX4, NRF2, SLC7A11, and ferritin heavy chain (FTH-1) levels increased (Figures 6A–I).

### 3.7 GA alleviated TBHP-induced ferroptosis in NP cells

**FIGURE 7:**
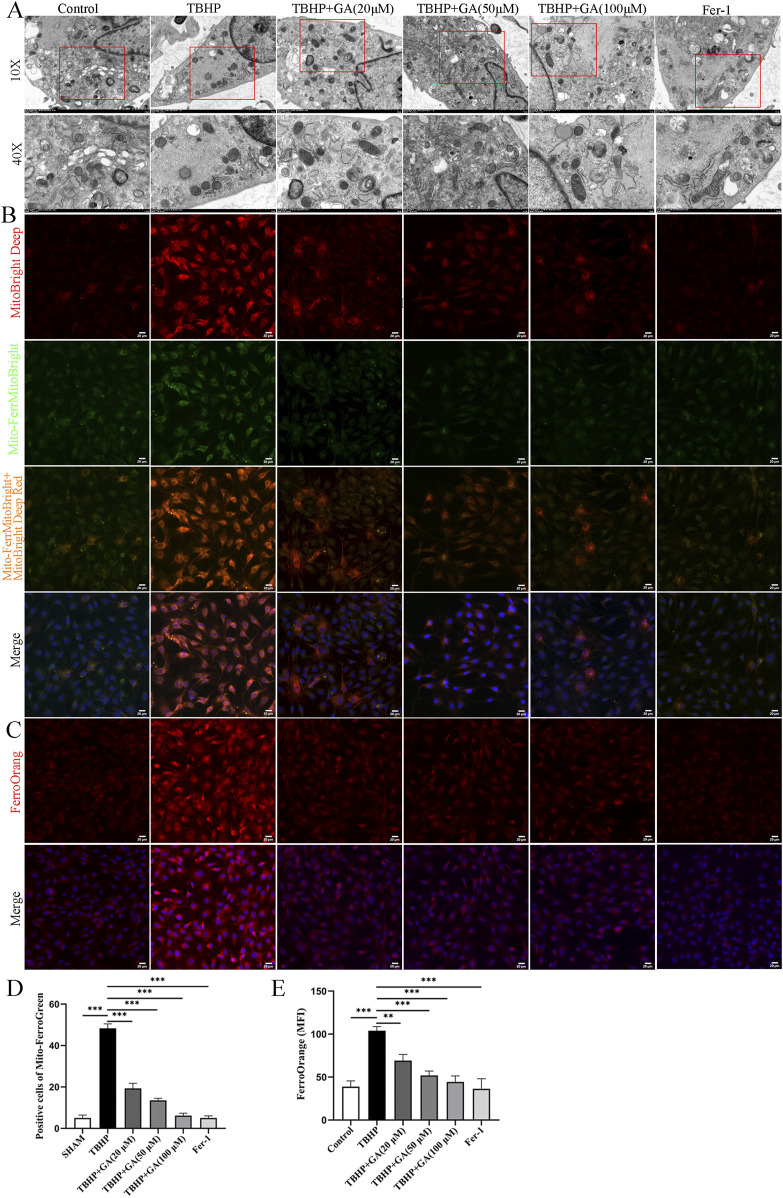
GA mitigates TBHP-induced ferroptosis in NP cells. **(A)** Representative images of the ultrastructure of mitochondria observed using transmission electron microscopy. **(B, C)** Representative immunofluorescence images of Mito-FerroGreen and FerroOrange staining in NP cells (scale bar = 20 μm). **(D)** Quantitative analysis of positive cells of Mito-FerroGreen staining. **(E)** Quantitative analysis of the mean fluorescence intensity of FerroOrange staining. ***p < 0.001.

To further study changes in mitochondrial morphology, TEM was used to evaluate mitochondrial ultrastructure. As previously reported, characteristic changes of ferroptosis were observed in TBHP -stimulated NP cells, including mitochondrial shrinkage and deformation, compression or reduction in size, rupture of the mitochondrial outer membrane, and reduction or disappearance of mitochondrial cristae. However, administration of GA can inhibit deleterious changes in mitochondrial morphology (Figure 7A). Iron overload is the hallmark and basis of ferroptosis, triggering a subsequent series of adverse reactions. After TBHP intervention in NP cells, both free Fe^2+^ and the green fluorescence intensity reflecting mitochondrial iron was significantly enhanced compared with the control group. Interestingly, compared with the TBHP stimulation group, all three concentrations of GA treatment attenuated the rise in mitochondrial iron (Figures 7B–E). From these findings, we can conclude that GA reduced intracellular and mitochondrial iron content while significantly reversing the ultrastructural and morphological changes of mitochondria in TBHP-stimulated NP cells.

## 4 Discussion

LBP negatively affects quality of life, with IDD being the main cause. Nonsteroidal anti-inflammatory drugs are currently commonly used to relieve pain symptoms (Samanta et al., 2023). However, these drugs are not effective in relieving IDD or stopping its progression (Francisco et al., 2022). Current studies indicate that ferroptosis is involved in the pathogenesis of IDD (Chen et al., 2022). Studying the molecular mechanisms of IDD and ferroptosis and developing drugs targeting specific molecules will be instructive in the future. GA has good effects on various degenerative diseases, involving anti-inflammatory and antioxidant stress processes (Moradi et al., 2021). Through network pharmacology and molecular docking, our study found that the main ferroptosis-related targets involved in GA treatment of IDD are P53, NRF2, and PPARg, which is consistent with previous research results (Dodson et al., 2019; Xie et al., 2016; Han et al., 2021).

After the intervertebral disc degenerates, the tissue morphology undergoes significant changes. The degradation of the ECM of the nucleus pulposus cells is the main pathological feature of IDD. Increased catabolism driven by inflammatory cytokines is an important factor leading to the degradation of ECM in the lumbar nucleus pulposus tissue (Zou et al., 2023). The main components of ECM include Collagen II and Aggrecan, and their degradation is closely related to the activity of ADAMTS4 and MMP3. This study showed that after acupuncture modeling of the intervertebral disc, the expression of ECM degradation proteins increased, resulting in pathological changes such as loss of NP tissue and disordered annulus fibrosus structure. GA treatment can inhibit the degradation of ECM in the NP cells of the degenerated intervertebral disc.

GPX4 is considered to be a major component of anti-peroxidative defense that maintains redox balance (Seibt et al., 2019). GPX4 combines with GSH to reduce lipid peroxidation of the cell membrane by consuming intracellular peroxidative substance (Ursini and Maiorino, 2020). Previous studies have shown that downregulation of GPX4 makes cells more sensitive to ferroptosis, while upregulation of GPX4 reduces cells’ sensitivity to ferroptosis (Liu et al., 2023). NRF2 is a major regulator of antioxidant and cytoprotective pathways (Dodson et al., 2019), and GSH-GPX4 is its downstream target in maintaining cellular redox balance (Xiang et al., 2024). NRF2 can regulate intracellular iron metabolism and is associated with oxidative stress and ferroptosis in macrophages (Anandhan et al., 2020). NRF2 can also inhibit ferroptosis by regulating SLC7A11 (Yuan et al., 2021). There is evidence that TP53 antagonizes ferroptosis in CRC cells by favoring the localization of DPP4 to the nuclear, enzymatically inactive pool (Xie et al., 2017). TP53 regulates ferroptosis through transcriptional or post-translational mechanisms and exhibits an inhibitory effect on SLC7A11 (Yang Y. et al., 2022). Excess P53 can regulate the expression of its downstream targets and promote the occurrence of ferroptosis. Genes related to iron metabolism have been confirmed to be key mediators in the ferroptosis process. Fe^3+^ enters cells through transferrinreceptor1 and is eventually reduced to Fe^2+^, which then mediates ROS production through the Fenton reaction. Downregulation of FTH-1 expression is the cause of iron overload in ferroptotic cells (Feng et al., 2023). Therefore, the regulatory effects of TP53 and NRF2 on ferroptosis may all be attributed to the effects on the expression of SLC7A11, FTH-1 and GPX4. This is consistent with the evidence we obtained in the expression of GPX4, NRF2, P53, FTH-1 and SLC7A11 proteins.

The main characteristic of ferroptosis is that cells produce excessive ROS during redox reactions, leading to mitochondrial damage. Previous studies have reported that oxidative stress is an activator of autophagy, cellular senescence, apoptosis, and abnormal ECM metabolism, all of which may induce mitochondrial dysfunction, thereby leading to excessive mitochondrial ROS production. The production of intracellular ROS and mitochondria triggers certain cellular responses, such as the removal of dysfunctional mitochondria and cells to protect adjacent mitochondria and cells from damage (Zorov et al., 2014). Therefore, we evaluated the intracellular ROS levels and mitochondrial morphology. Our study showed that GA was able to reverse the excessive ROS production and mitochondrial damage caused by TBHP. Lipid peroxidation driven by ROS can destroy the composition and structure of cell membranes and produce large amounts of lethal MDA. MDA is a lipid peroxidation metabolite, and its excessive accumulation can lead to changes in cell membrane fluidity and permeability, thereby affecting the structure and function of cells (Ru et al., 2024). Our results showed that TBHP significantly increased MDA, free Fe^2+^ and mitochondrial Fe^2+^. However, GA treatment reversed these changes. This shows that GA can inhibit ferroptosis in NP cells by inhibiting lipid peroxidation and improving oxidative stress.

GA is a natural product extracted from a variety of traditional Chinese medicines and has excellent antioxidant functions. It has been shown to inhibit P2X7 receptor-mediated ferroptosis in rat spinal cord and to inhibit ferroptosis-related genes that predict overall survival in colorectal cancer patients (Yang et al., 2023). Based on literature research and prediction results from network pharmacology, we further revealed the mechanism by which GA inhibits ferroptosis in IDD. In the rat tail puncture-induced degeneration model, GA can not only upregulate NRF2 and downregulate P53 in the NP tissue, but also mitigate IDD. Under the action of GA, Fe^2+^, Liperfluo and ROS are inhibited in the NP cells induced by TBHP. The expression of GPX4, NRF2, P53, FTH-1 and SLC7A11 proteins is consistent with the evidence from previous studies.

Previous studies have found that GA can be used to treat tumors and various degenerative diseases by inhibiting oxidative stress by weakening the inflammatory response. This study reports the ROS metabolism process of GA, its novel preventive effect on IDD and its inhibitory effect on ferroptosis of NP cells.

## 5 Conclusion

In conclusion, this study provides evidence for a role of ferroptosis in disc degeneration. It identified multiple indicators related to ferroptosis in the treatment of IDD by GA through network pharmacology. It was confirmed that GA has a certain inhibitory effect on IDD ferroptosis *in vitro* and *in vivo*, which may be achieved by regulating P53 and Nrf2. The potential of the natural product GA as a therapeutic agent for IDD was demonstrated.

## Data Availability

The raw data supporting the conclusions of this article will be made available by the authors, without undue reservation.

## References

[B1] Anandhan A. Dodson M. Schmidlin C. J. Liu P. Zhang D. D. (2020). Breakdown of an ironclad defense system: the critical role of NRF2 in mediating ferroptosis. Cell Chem. Biol. 27, 436–447. 10.1016/j.chembiol.2020.03.011 32275864 PMC7597851

[B2] Antoniou J. Steffen T. Nelson F. Winterbottom N. Hollander A. P. Poole R. A. (1996). The human lumbar intervertebral disc: evidence for changes in the biosynthesis and denaturation of the extracellular matrix with growth, maturation, ageing, and degeneration. J. Clin. Invest. 98, 996–1003. 10.1172/JCI118884 8770872 PMC507515

[B6] Chen J. Yang X. Feng Y. Li Q. Ma J. Wang L. (2022). Targeting ferroptosis holds potential for intervertebral disc degeneration therapy. Cells-Basel 11, 3508. 10.3390/cells11213508 PMC9653619 36359904

[B7] Chen Q. Qian Q. Xu H. Zhou H. Chen L. Shao N. (2024). Mitochondrial-targeted metal-phenolic nanoparticles to attenuate intervertebral disc degeneration: alleviating oxidative stress and mitochondrial dysfunction. Acs Nano 18, 8885–8905. 10.1021/acsnano.3c12163 38465890

[B8] Dixon S. J. Lemberg K. M. Lamprecht M. R. Skouta R. Zaitsev E. M. Gleason C. E. (2012). Ferroptosis: an iron-dependent form of nonapoptotic cell death. Cell 149, 1060–1072. 10.1016/j.cell.2012.03.042 22632970 PMC3367386

[B9] Dixon S. J. Stockwell B. R. (2014). The role of iron and reactive oxygen species in cell death. Nat. Chem. Biol. 10, 9–17. 10.1038/nchembio.1416 24346035

[B10] Dodson M. Castro-Portuguez R. Zhang D. D. (2019). NRF2 plays a critical role in mitigating lipid peroxidation and ferroptosis. Redox Biol. 23, 101107. 10.1016/j.redox.2019.101107 30692038 PMC6859567

[B11] Dong H. Xia Y. Jin S. Xue C. Wang Y. Hu R. (2021). Nrf2 attenuates ferroptosis-mediated IIR-ALI by modulating TERT and SLC7A11. Cell Death Dis. 12, 1027. 10.1038/s41419-021-04307-1 34716298 PMC8556385

[B12] Feng C. Liu H. Yang M. Zhang Y. Huang B. Zhou Y. (2016). Disc cell senescence in intervertebral disc degeneration: causes and molecular pathways. Cell Cycle 15, 1674–1684. 10.1080/15384101.2016.1152433 27192096 PMC4957599

[B13] Feng Q. Yang Y. Qiao Y. Zheng Y. Yu X. Liu F. (2023). Quercetin ameliorates diabetic kidney injury by inhibiting ferroptosis via activating Nrf2/HO-1 signaling pathway. Am. J. Chin. Med. 51, 997–1018. 10.1142/S0192415X23500465 37046368

[B14] Francisco V. Pino J. Gonzalez-Gay M. A. Lago F. Karppinen J. Tervonen O. (2022). A new immunometabolic perspective of intervertebral disc degeneration. Nat. Rev. Rheumatol. 18: 47–60. 10.1038/s41584-021-00713-z 34845360

[B15] Han B. Zhu K. Li F. C. Xiao Y. X. Feng J. Shi Z. L. (2008). A simple disc degeneration model induced by percutaneous needle puncture in the rat tail. Spine (Phila Pa 1976) 33, 1925–1934. 10.1097/BRS.0b013e31817c64a9 18708924

[B16] Han L. Bai L. Qu C. Dai E. Liu J. Kang R. (2021). PPARG-mediated ferroptosis in dendritic cells limits antitumor immunity. Biochem. Biophys. Res. Commun. 576, 33–39. 10.1016/j.bbrc.2021.08.082 34478917

[B17] Hong Z. Tang P. Liu B. Ran C. Yuan C. Zhang Y. (2021). Ferroptosis-related genes for overall survival prediction in patients with colorectal cancer can be inhibited by gallic acid. Int. J. Biol. Sci. 17, 942–956. 10.7150/ijbs.57164 33867820 PMC8040315

[B18] Huang Y. Chen J. Jiang T. Zhou Z. Lv B. Yin G. (2017). Gallic acid inhibits the release of ADAMTS4 in nucleus pulposus cells by inhibiting p65 phosphorylation and acetylation of the NF-κB signaling pathway. Oncotarget 8, 47665–47674. 10.18632/oncotarget.17509 28512264 PMC5564596

[B19] Ingold I. Berndt C. Schmitt S. Doll S. Poschmann G. Buday K. (2018). Selenium utilization by GPX4 is required to prevent hydroperoxide-induced ferroptosis. Cell 172, 409–422.e21. 10.1016/j.cell.2017.11.048 29290465

[B20] Jiang X. Stockwell B. R. Conrad M. (2021). Ferroptosis: mechanisms, biology and role in disease. Nat. Rev. Mol. Cell Biol. 22, 266–282. 10.1038/s41580-020-00324-8 33495651 PMC8142022

[B21] Kang R. Kroemer G. Tang D. (2019). The tumor suppressor protein p53 and the ferroptosis network. Free Radic. Biol. Med. 133, 162–168. 10.1016/j.freeradbiomed.2018.05.074 29800655 PMC6251771

[B22] Keorochana G. Johnson J. S. Taghavi C. E. Liao J. C. Lee K. B. Yoo J. H. (2010). The effect of needle size inducing degeneration in the rat caudal disc: evaluation using radiograph, magnetic resonance imaging, histology, and immunohistochemistry. Spine J. 10, 1014–1023. 10.1016/j.spinee.2010.08.013 20970740

[B23] Li Y. Chen L. Gao Y. Zou X. Wei F. (2022). Oxidative stress and intervertebral disc degeneration: pathophysiology, signaling pathway, and therapy. Oxid. Med. Cell Longev. 1984742. 10.1155/2022/1984742 36262281 PMC9576411

[B24] Liu Y. Wan Y. Jiang Y. Zhang L. Cheng W. (2023). GPX4: the hub of lipid oxidation, ferroptosis, disease and treatment. Biochim. Biophys. Acta Rev. Cancer 1878, 188890. 10.1016/j.bbcan.2023.188890 37001616

[B25] Lu S. Song Y. Luo R. Li S. Li G. Wang K. (2021). Ferroportin-dependent iron homeostasis protects against oxidative stress-induced nucleus pulposus cell ferroptosis and ameliorates intervertebral disc degeneration *in vivo* . Oxid. Med. Cell Longev. 2021, 6670497. 10.1155/2021/6670497 33628376 PMC7889334

[B26] Ma F. Gong X. Zhou X. Zhao Y. Li M. (2015). An UHPLC-MS/MS method for simultaneous quantification of gallic acid and protocatechuic acid in rat plasma after oral administration of Polygonum capitatum extract and its application to pharmacokinetics. J. Ethnopharmacol. 162, 377–383. 10.1016/j.jep.2014.12.044 25557034

[B27] Moradi A. Abolfathi M. Javadian M. Heidarian E. Roshanmehr H. Khaledi M. (2021). Gallic acid exerts nephroprotective, anti-oxidative stress, and anti-inflammatory effects against diclofenac-induced renal injury in malerats. Arch. Med. Res. 52, 380–388. 10.1016/j.arcmed.2020.12.005 33358172

[B28] Oichi T. Taniguchi Y. Oshima Y. Tanaka S. Saito T. (2020). Pathomechanism of intervertebral disc degeneration. JOR Spine 3, e1076. 10.1002/jsp2.1076 32211588 PMC7084053

[B29] Raj P. P. (2008). Intervertebral disc: anatomy-physiology-pathophysiology-treatment. Pain Pract. 8, 18–44. 10.1111/j.1533-2500.2007.00171.x 18211591

[B30] Risbud M. V. Shapiro I. M. (2014). Role of cytokines in intervertebral disc degeneration: pain and disc content. Nat. Rev. Rheumatol. 10, 44–56. 10.1038/nrrheum.2013.160 24166242 PMC4151534

[B31] Ru Y. Luo Y. Liu D. Huang Q. Zhou X. Linghu M. (2024). Isorhamnetin alleviates ferroptosis-mediated colitis by activating the NRF2/HO-1 pathway and chelating iron. Int. Immunopharmacol. 135, 112318. 10.1016/j.intimp.2024.112318 38795598

[B32] Sakai D. Grad S. (2015). Advancing the cellular and molecular therapy for intervertebral disc disease. Adv. Drug Deliv. Rev. 84, 159–171. 10.1016/j.addr.2014.06.009 24993611

[B33] Samanta A. Lufkin T. Kraus P. (2023). Intervertebral disc degeneration-Current therapeutic options and challenges. Front. Public Health 11, 1156749. 10.3389/fpubh.2023.1156749 37483952 PMC10359191

[B34] Seibt T. M. Proneth B. Conrad M. (2019). Role of GPX4 in ferroptosis and its pharmacological implication. Free Radic. Biol. Med. 133: 144–152. 10.1016/j.freeradbiomed.2018.09.014 30219704

[B35] Stockwell B. R. Friedmann A. J. Bayir H. Bush A. I. Conrad M. Dixon S. J. (2017). Ferroptosis: a regulated cell death nexus linking metabolism, redox biology, and disease. Cell 171, 273–285. 10.1016/j.cell.2017.09.021 28985560 PMC5685180

[B36] Suyama K. Sakai D. Watanabe M. (2022). The role of IL-17-mediated inflammatory processes in the pathogenesis of intervertebral disc degeneration and herniation: a comprehensive review. Front. Cell Dev. Biol. 10, 857164. 10.3389/fcell.2022.857164 35309927 PMC8927779

[B37] Tang H. M. Cheung P. (2019). Gallic acid triggers iron-dependent cell death with apoptotic, ferroptotic, and necroptotic features. Toxins (Basel) 11, 492. 10.3390/toxins11090492 31455047 PMC6783835

[B38] Ursini F. Maiorino M. (2020). Lipid peroxidation and ferroptosis: the role of GSH and GPx4. Free Radic. Biol. Med. 152, 175–185. 10.1016/j.freeradbiomed.2020.02.027 32165281

[B39] Wang W. Jing X. Du T. Ren J. Liu X. Chen F. (2022). Iron overload promotes intervertebral disc degeneration via inducing oxidative stress and ferroptosis in endplate chondrocytes. Free Radic. Biol. Med. 190, 234–246. 10.1016/j.freeradbiomed.2022.08.018 35981695

[B40] Wang Z. Xia Q. Liu X. Liu W. Huang W. Mei X. (2018). Phytochemistry, pharmacology, quality control and future research of Forsythia suspensa (Thunb.) Vahl: a review. J. Ethnopharmacol. 210, 318–339. 10.1016/j.jep.2017.08.040 28887216

[B41] Wu P. H. Kim H. S. Jang I. T. (2020). Intervertebral disc diseases part 2: a review of the current diagnostic and treatment strategies for intervertebral disc disease. Int. J. Mol. Sci. 21, 2135. 10.3390/ijms21062135 32244936 PMC7139690

[B42] Xiang Y. Song X. Long D. (2024). Ferroptosis regulation through Nrf2 and implications for neurodegenerative diseases. Arch. Toxicol. 98, 579–615. 10.1007/s00204-023-03660-8 38265475 PMC10861688

[B43] Xie T. Bai Z. Chen Z. Liang H. Liu T. Lam L. K. (2023). Inhibition of ferroptosis ameliorates hypertensive nephropathy through p53/Nrf2/p21 pathway by Taohongsiwu decoction: based on network pharmacology and experimental validation. J. Ethnopharmacol. 312, 116506. 10.1016/j.jep.2023.116506 37086874

[B44] Xie Y. Hou W. Song X. Yu Y. Huang J. Sun X. (2016). Ferroptosis: process and function. Cell Death Differ. 23, 369–379. 10.1038/cdd.2015.158 26794443 PMC5072448

[B45] Xie Y. Zhu S. Song X. Sun X. Fan Y. Liu J. (2017). The tumor suppressor p53 limits ferroptosis by blocking DPP4 activity. Cell Rep. 20, 1692–1704. 10.1016/j.celrep.2017.07.055 28813679

[B46] Yang R. Shi L. Si H. Hu Z. Zou L. Li L. (2023). Gallic acid improves comorbid chronic pain and depression behaviors by inhibiting P2X7 receptor-mediated ferroptosis in the spinal cord of rats. Acs Chem. Neurosci. 14, 667–676. 10.1021/acschemneuro.2c00532 36719132

[B47] Yang R. Z. Xu W. N. Zheng H. L. Zheng X. F. Li B. Jiang L. S. (2021). Involvement of oxidative stress-induced annulus fibrosus cell and nucleus pulposus cell ferroptosis in intervertebral disc degeneration pathogenesis. J. Cell. Physiol. 236, 2725–2739. 10.1002/jcp.30039 32892384 PMC7891651

[B48] Yang W. Wang Y. Zhang C. Huang Y. Yu J. Shi L. (2022a). Maresin1 protect against ferroptosis-induced liver injury through ROS inhibition and Nrf2/HO-1/GPX4 activation. Front. Pharmacol. 13, 865689. 10.3389/fphar.2022.865689 35444546 PMC9013935

[B49] Yang Y. Ma Y. Li Q. Ling Y. Zhou Y. Chu K. (2022b). STAT6 inhibits ferroptosis and alleviates acute lung injury via regulating P53/SLC7A11 pathway. Cell Death Dis. 13, 530. 10.1038/s41419-022-04971-x 35668064 PMC9169029

[B50] Yuan Y. Zhai Y. Chen J. Xu X. Wang H. (2021). Kaempferol ameliorates oxygen-glucose deprivation/reoxygenation-induced neuronal ferroptosis by activating nrf2/slc7a11/GPX4 Axis. Biomolecules 11, 923. 10.3390/biom11070923 34206421 PMC8301948

[B51] Zhang L. Zhang J. Jin Y. Yao G. Zhao H. Qiao P. (2021). Nrf2 is a potential modulator for orchestrating iron homeostasis and redox balance in cancer cells. Front. Cell Dev. Biol. 9, 728172. 10.3389/fcell.2021.728172 34589492 PMC8473703

[B52] Zhang P. Rong K. Guo J. Cui L. Kong K. Zhao C. (2023). Cynarin alleviates intervertebral disc degeneration via protecting nucleus pulposus cells from ferroptosis. Biomed. Pharmacother. 165, 115252. 10.1016/j.biopha.2023.115252 37536034

[B54] Zorov D. B. Juhaszova M. Sollott S. J. (2014). Mitochondrial reactive oxygen species (ROS) and ROS-induced ROS release. Physiol. Rev. 94, 909–950. 10.1152/physrev.00026.2013 24987008 PMC4101632

[B55] Zou X. Zhang X. Han S. Wei L. Zheng Z. Wang Y. (2023). Pathogenesis and therapeutic implications of matrix metalloproteinases in intervertebral disc degeneration: a comprehensive review. Biochimie 214, 27–48. 10.1016/j.biochi.2023.05.015 37268183

